# Substance Use, Highly Active Antiretroviral Therapy, and Liver Enzymes: Evidence From a Cross-Sectional Study of HIV-Infected Adult Patients Without Comorbidities on HAART in the University of Port Harcourt Teaching Hospital

**DOI:** 10.3389/frph.2021.664080

**Published:** 2021-06-28

**Authors:** Chinwe F. Anyanwu, Tamuno-Olobo JohnBull, Ibe M. Usman, Eric O. Aigbogun, Joy Ochai, Ahmed H. Qasem, Shadi S. Alkhayyat, Athanasios Alexiou, Gaber El-Saber Batiha

**Affiliations:** ^1^Department of Pharmacology, Faculty of Basic Clinical Sciences, University of Port Harcourt, Port Harcourt, Nigeria; ^2^Department of Human Anatomy, Faculty of Basic Medical Sciences, Niger Delta University, Amassoma, Nigeria; ^3^Department of Anatomy, Faculty of Biomedical Sciences, Kampala International University, Kampala, Uganda; ^4^Department of Public Health Science, Faculty of Science and Technology, Cavendish University, Kampala, Uganda; ^5^Human Anatomy Department, Faculty of Basic Medical Sciences, Ahmadu Bello University, Zaria, Nigeria; ^6^Laboratory Medicine Department, Faculty of Applied Medical Sciences, Umm Al-Qura University, Mecca, Saudi Arabia; ^7^Department of Internal Medicine, Faculty of Medicine, King Abdulaziz University, Jeddah, Saudi Arabia; ^8^Novel Global Community Educational Foundation, Hebersham, NSW, Australia; ^9^AFNP Med Austria, Wien, Austria; ^10^Department of Pharmacology and Therapeutics, Faculty of Veterinary Medicine, Damanhour University, Damanhour, Egypt

**Keywords:** SEM, substance use, enzyme levels, HAART, HIV-infected adults

## Abstract

This study applied a structural equation modeling (SEM) to evaluate the role of substance use (alcohol, smoking, and trado-medicine use) to changes in the liver enzymes (AST, ALT, and ALP) levels in HIV-infected adult patients on a highly active antiretroviral treatment (HAART) for not <1 year. The study was a cross-sectional, part of a randomized comparative trial (Ref: UPH/CEREMAD/REC/19), involving 129 (46 males and 83 females) HIV-infected adult patients. Liver enzyme levels were determined from analyzed blood samples using the Clinical Chemistry Analyser (VS10) manufactured by Vitro Scient, while the study determined substance use using a reliable (Cronbach alpha = 0.805) rapid-exploratory survey questionnaire. Liver enzyme values were further categorized into: normal or abnormal using normal reference ranges (ALT = 7–55 U/L, AST = 8–48 U/L, and ALP = 40–129 U/L). STATGRAPHICS V16.1.11 (StatPoint Tech., Inc.) and SPSS (IBM® Amos V21.0.0, USA) were used to analyze the data. Among the HIV-HAART patients, 27.9% were alcohol users, 20.9% smokers, and 20.1% trado-medicine users. In addition, ALP (71.3%) abnormality was higher than ALT (34.9%) and AST (28.7%). The result from the SEM provided only a partial support for our hypotheses of direct substance use effects on the liver enzyme levels and abnormalities; with a direct association of alcohol with an elevated AST (*b* = 0.170, *p* = 0.05) and smoking with a higher AST (*b* = 0.484, *p* < 0.01) and ALT (*b* = 0.423, *p* < 0.01) values. Trado-medicine use was not directly associated with enzyme elevation and abnormality. In conclusion, ALP abnormality was the most common, and there is a close association between an elevated ALT and AST, with or without an elevated ALP. The study found that HIV-HAART patients who drink or smoke will have at least one or more abnormal transaminases. The possible explanation to the increased risk among HIV-HAART patients could be associated with the metabolic pressures and supra-additive effects on the livers.

## Introduction

The human immunodeficiency virus (HIV) is from the genus Lentivirus within the family of Retroviridae, subfamily Orthoretrovirinae (1, 2). HIV is classified into types 1 and 2 (HIV-1, HIV-2), based on the genetic characteristics and differences in the viral antigens (2–6). The origin of HIV-2 has long been resolved (7), and it remains as the most common type of HIV among individuals from west Africa (8). In 1989, a closely related simian immunodeficiency virus (SIV) was found in a monkey, the sooty mangabey (*Cercocebus atys*), whose natural range is in west Africa (9). A virus closely related to HIV-1 was first reported in 1989; this virus, SIVcpz, was found in two captive chimpanzees (*Pan troglodytes*) in Gabon (10).

The Human Immunodeficiency Virus/Acquired Immunodeficiency Syndrome (HIV/AIDS) is a global health problem; over 70 million people have been infected with HIV, 35 million have died, and 36.7 million people currently live with the disease (11). HIV continues to spread rapidly, with more than 1.7 (1.2–2.2) million new infections in 2019 (12), and Sub-Saharan Africa is the hardest-hit region in the world, with more than two-thirds of all people living with HIV globally (13). The introduction of behavioral interventions, even after a successful scaling up to achieve a sufficient coverage in many populations, have not resulted in significant declines in HIV incidence (14), and it will take years to develop highly effective HIV-preventive vaccines (14–19). For more than a decade, the increasingly well-tolerated highly active antiretroviral therapy [HAART, which incorporates three or more antiretroviral therapy (ART) medications] has dramatically changed HIV-associated morbidity and mortality, and improved the quality of life of HIV-infected individuals (20–26).

**Figure 1:**
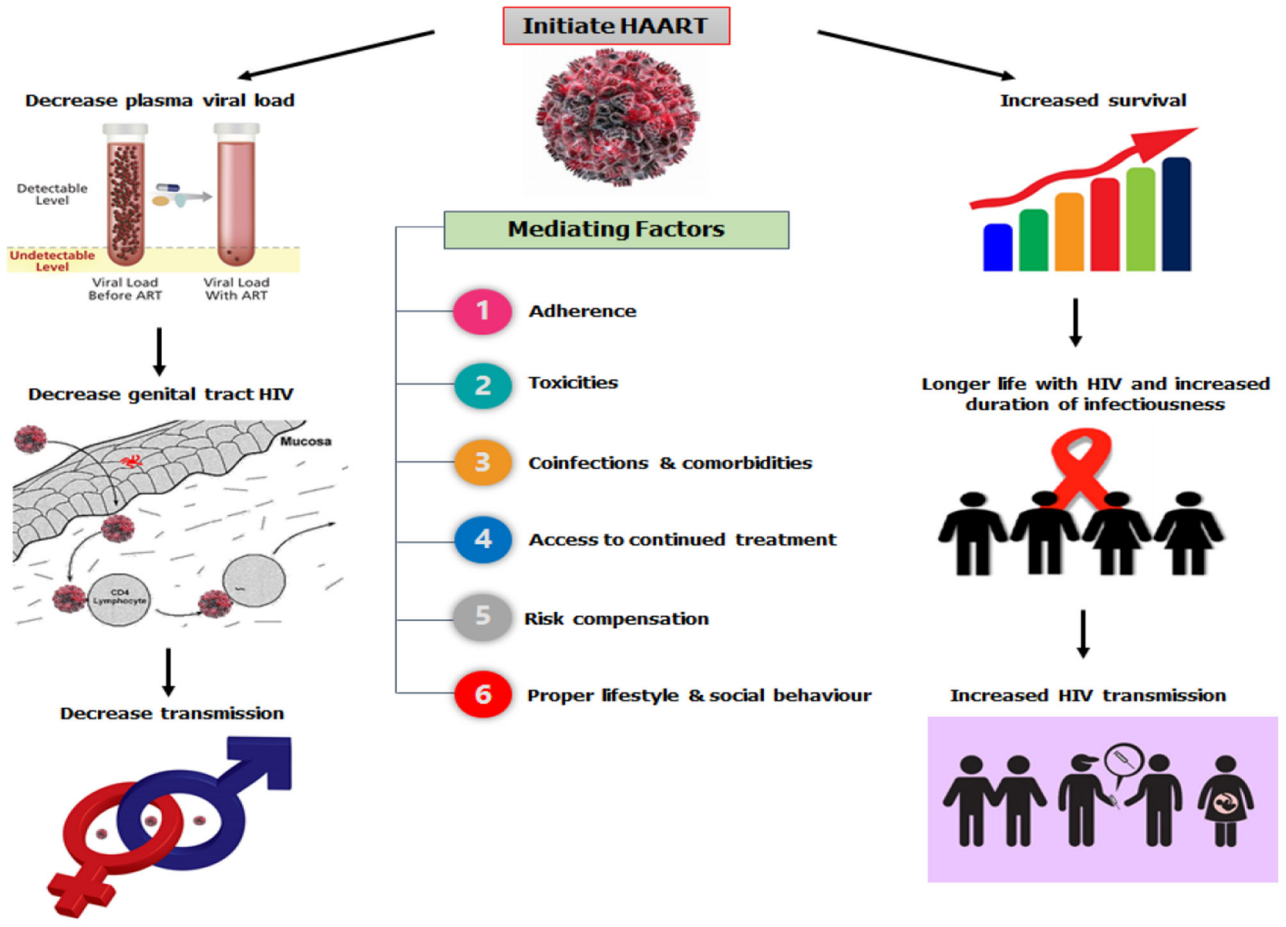
HAART and HIV transmission dynamics.

HAART has dramatically decreased mother-to-child HIV transmission (27–29); and convincingly prevented the sexual transmission of HIV via reductions in genital tract HIV concentrations in individuals who are already infected (30–32), or as a pre- or post-exposure prophylaxis for uninfected people exposed to HIV (30, 33–35). The potential effects of HAART on HIV are shown in Figure 1 (26).

The effects of HAART initiation can manifest differently in diverse social settings because sexual behavior (36–39) and substance use (40–42) involve concerns over pleasure and procreation (36, 37). Such behaviors are associated with poorer outcomes in the management of HIV (43–45) and the continued proliferation of new infections (46, 47). Unfortunately, the use of antiretroviral therapy has not been without issue (48), as studies have reported systemic toxicity (both short- and long-term) following its commencement (49–51) or a change (52–55) of HAART. One of the most affected systems is the hepatic system (40–42, 49, 50), which causes abnormalities in the liver enzymes (ALT, AST, and ALP). Studies have suggested that when patients are on HAART, in the nearest future, they present with abnormal hepatic enzyme levels (53, 54, 56, 57), which may even lead to liver failure (58, 59). However, co-infection with hepatitis B or C virus (48, 60–63) and tuberculosis treatment (64, 65) could induce liver toxicity, with a significant elevation in the ALT and AST levels (66).

Additionally, the degree of hepatic damage in HIV patients on HAART is significantly associated with age, gender, lifestyle, obesity, and herbal medications (40–42). Substance abuse is prevalent among people with HIV (67, 68), and it contributes to poor health outcomes (43, 68). This is because of the risk or severity of substance-related toxicities, and arising drug-substance interactions are often unpredictable (69). Studies have found that alcohol consumption and smoking has independent and supra-additive effects on liver enzymes (70–73), especially when a metabolic abnormality is present (72). Another study found that smoking may enhance the effects of alcohol on liver cell injury in heavy drinkers (73). Substance use and abuse in HIV-patients is known to cause a reduced adherence to ART (44, 45).

The concerns about the extent to which substance use could influence liver enzyme in HIV-patients who are already on HAART known to deleteriously affect the liver is genuine. Therefore, against this background, this study evaluated the direct association between substance use (alcohol drinking, smoking, and trado-medicine use) and the liver enzyme levels of HIV-patients without comorbidities on HAART in UPTH using a structural equation modeling (SEM).

## Methods

### Study Setting and Population

**Figure 2:**
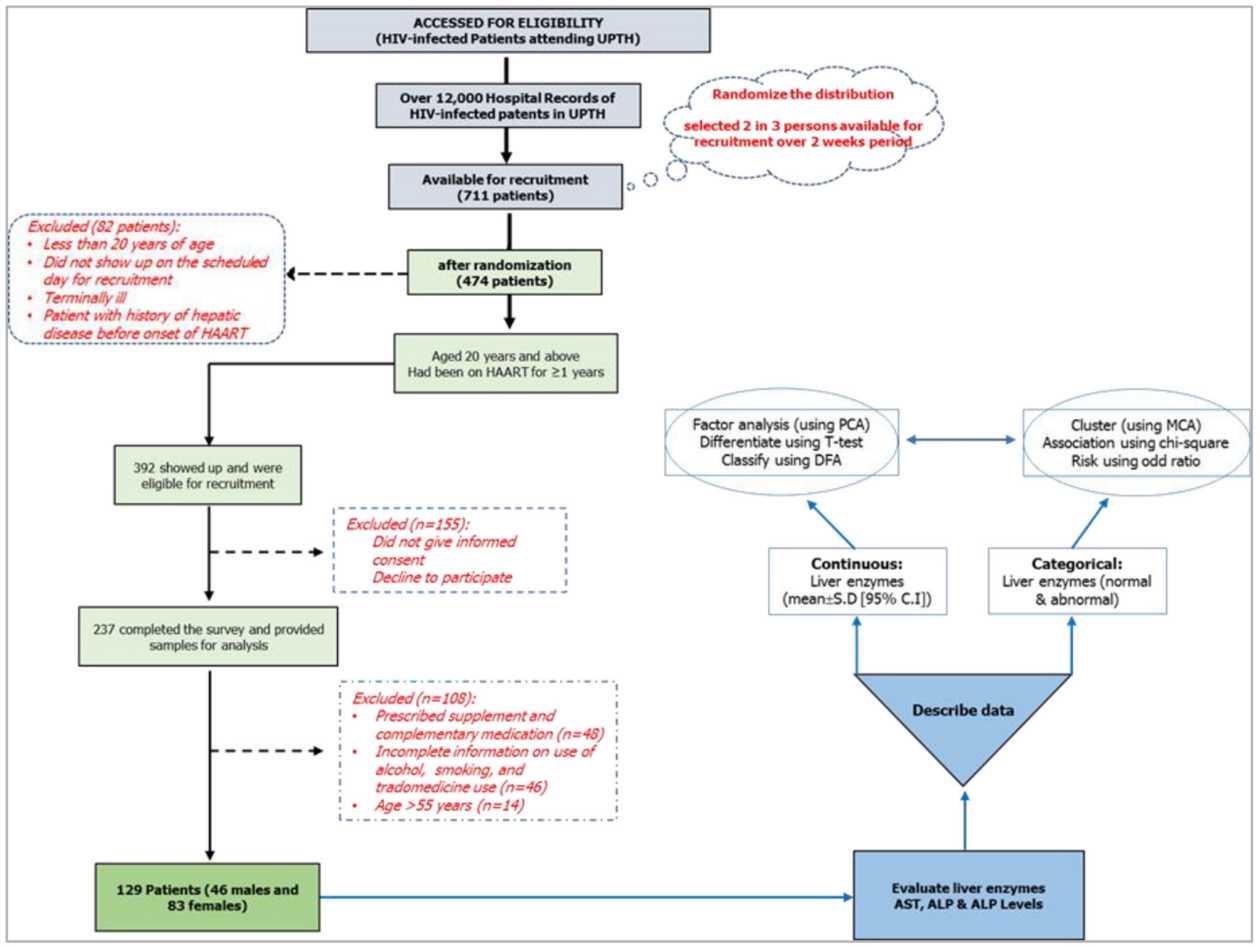
Study design and research lifecycle.

This study was conducted between June 2016 and November 2018 using HIV-infected patients at the University of Port Harcourt. The HIV clinic registered about 12,000 HIV patients who received antiretrovirals and counseling. Out of the 12,000 registered patients, the data for 711 HIV-infected patients were reviewed, and 474 patients were approached for the study; for which, 392 patients met the criteria and were physically present for the recruitment. Out of the 392 that were included in the study, 155 were excluded for declining to participate or refusal to provide a written/signed informed consent (Figure 2); thus, only 237 patients filled the survey and provided samples for biochemical analysis within the study period. The inclusion criteria included patients: (i) diagnosed with HIV infection and are undergoing HAART; (ii) aged >20 years; and (iii) continuing HAART (≥12 months) with records indicating that their baseline serum ALT, AST, and ALP levels were within normal ranges (ALT = 7–55 U/L; AST = 8–48 U/L; ALP = 40–129 U/L) before the commencement of HAART. Patients that did not meet the inclusion criteria were excluded: patients with acute HIV infection, co-infections, or severe, life-threatening complications; who were pregnant; with autoimmune diseases; incomplete data; and >55 years of age (74–76). The study obtained ethical approval (reference number UPH/CEREMAD/REC/18) from the Research Ethics Committee of the University of Port Harcourt, and written and signed informed consent from the patients (77).

### Research Design

This study was designed as a cross-sectional study, part of a 2016 randomized control study (CEREMAD) (74, 78), involving HIV-positive patients on HAART for not <1 year (12 months) with a CD4 count not less than the critical value of 200 cells/mm^3^ and enzyme levels within normal range prior to the commencement of HAART (which was determined as at the start of 2016). Personal information such as age, sex, and substance use (alcohol, smokes, trado-medicine) were collected. The study inquired about the pregnancy status for the females, the start date of HAART, and co-infections (such as pulmonary tuberculosis, hepatitis B and C, STIs) from their hospital folders and pre-tested questionnaires, and these were used as the selection criteria for the participants (Figure 2).

**Figure 3:**
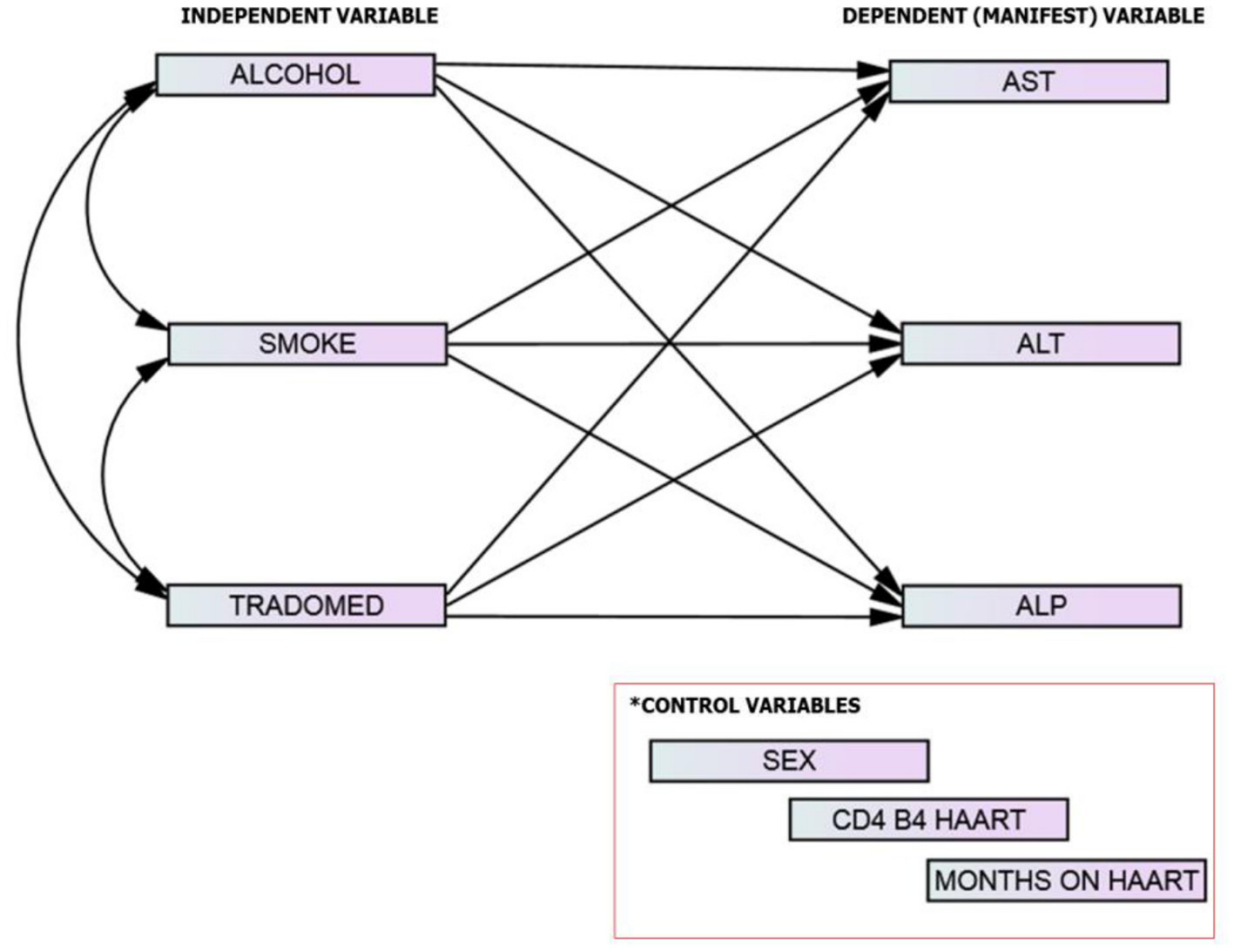
The study model/framework.

The conceptual framework was designed to evaluate (using direct effect path models) the relationships between substance use and liver enzymes. The exogenous (independent) variables are described as substance use (alcohol use, smoking, trado-medicine use) while the endogenous (dependent; manifest variables) were the liver enzymes levels (ALT, AST, and ALP). The control variables are sex, CD4 on starting HAART, and months on HAART. The study described the manifest variables as both continuous and categorical variables (normal/abnormal) (Figure 3).

Based on the theoretical constructs of the model framework and empirical evidences (70–73), we hypothesized that in HIV-infected adults without comorbidities on HAART, (1) substance use is associated with an increase in liver enzymes levels and (2) there is a direct association between substance use and abnormal liver enzymes.

### Estimating Liver Enzymes Levels and Substance Use

Venous blood samples were collected and serum levels of aspartate aminotransferase [AST], alanine transaminase [ALT], and alkaline phosphatase [ALP] were evaluated using the Clinical Chemistry Analyser (VS10) manufactured by Vitro Scient. The machine utilizes the Beer-lambert's law (that is, the linear relationship between absorbance and concentration of an absorbing species) in estimating the enzyme levels (74). Normal range values were established in units per liter as follows: ALT (7–55 U/L), AST (8–48 U/L), and ALP (40–129 U/L) (74, 75). Substance use with regard to alcoholism, smoking, and traditional herbal use while on HAART was determined by a short-rapid exploratory survey using a closed-ended questionnaire.

### Data Management and Analysis

Out the 237 patients that filled the survey and provided samples for analysis, 108 were not part of the analysis for the following reasons: on a prescribed supplement and complementary medication (*n* = 48); provided incomplete information on use of alcohol, smoking, and trado-medicine (*n* = 46); and age >55 years (*n* = 14). A total of 129 data were managed (clean and code) in Microsoft Excel 2016. The obtained ALT, AST, and ALP values were categorized into normal (within normal range) and abnormal (outside normal range) levels (75). The dataset used in this study was deposited in the Harvard Dataverse repository (76).

The cleaned data were analyzed using the STATGRAPHICS centurion CVI version 16.1.11 (StatPoint Tech., Inc.) and Statistics Package for Social Science (SPSS)–(IBM® Amos V21.0.0, USA). The descriptive statistics were performed for continuous data and represented as mean (S.D) while frequencies (%) represented the categorical data. Fisher's Chi-square (Yates correction) analysis evaluated the sex-associated differences in the distribution, and test of mean differences in the liver enzyme levels. The confidence level was set at 95%, and a *P* < 0.05 was considered significant.

SPSS–Amos structural equation modeling (SEM) was used to evaluate the direct effects of substance use on the enzyme levels (alcohol, smoking, and trado-medicine use) and the relationship between the predictor variables, while controlling for sex, CD4 cell count, and duration of HAART. To ensure an effective analytical path, all categorical variables were in binary forms, and the model controlled for sex, CD4 on starting HAART, and duration on HAART. To account for the non-normal distribution of the model variables, we used the maximum likelihood with robust standard errors (MLR) as the estimator. We chose two fit indices to assess the fit of the models: (i) the goodness of fit index (GFI) under generalized least squares (GLS) (79) and (ii) Bentler's comparative fit index (CFI) (80). Values between 0.90 and 1.0 on Bentler's CFI and value ≥0.95 on the GFI indicate that the model provides a good fit to the data (81). The study also determined the standardized and unstandardized direct effects of the exogenous variables on the manifest variables.

## Results

**Figure 4:**
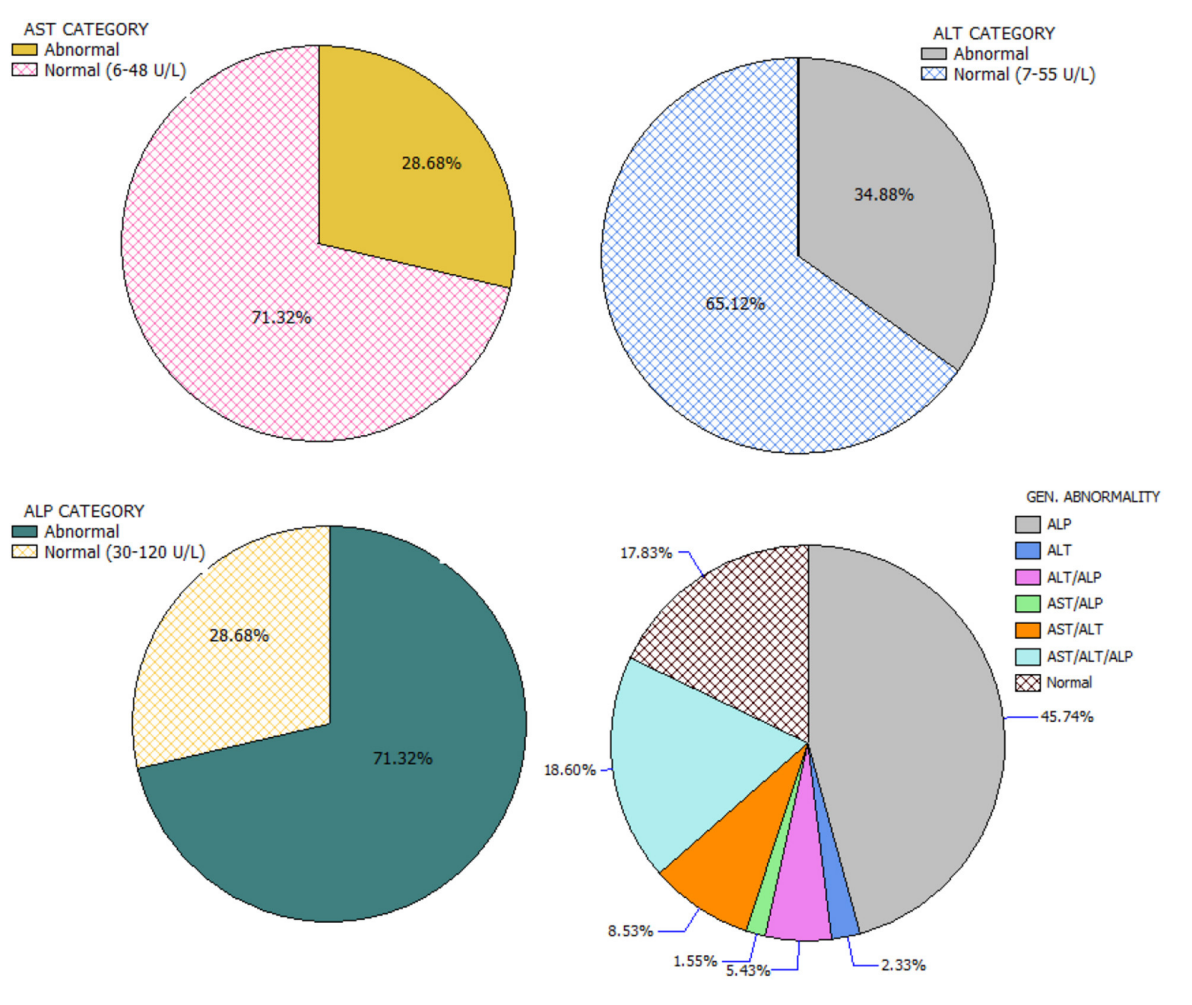
Categorization and distribution of enzyme abnormalities.

The study received data of 129 HIV-infected adults (46 males and 83 females) who fulfilled the minimum requirement for participation in the study. Among the participants, alcohol users were 27.9%, smokers 20.9%, and trado-medicine users 20.1% (Table 1). The proportion with different abnormalities were as follows: AST (28.7%), ALT (34.9%), and ALP (71.3%) (Figure 4). The mean CD4 (in cells/mm^3^) prior to the commencement of HAART was 290.54 ± 95.41 for males and 301.22 ± 89.57 females; with a population median of 270.99 (range = 200.0–611.0). The mean duration on HAART (in months) for males was 17.25 ± 4.40 and females 18.26 ± 4.68; with a population median of 16.99 (range = 12–27) (Table 2). There was no significant association between substance use with sex (*P* > 0.05) (Table 1). The median CD4 cells count and duration on HAART were not significantly different between the categories for substance use (*P* > 0.05) (Table 2). For the distribution of enzyme level across the substance use groups, see Figures A1.1–A1.3 in Supplementary Material.

**Table 1:** Substance use [alcohol use, smoking, and trado-medicine (herbal medicines)] among the HIV-infected patients on HAART.

**Variables**		**Sex**		**Total**	**^**Y**^Chi-square (*P*-value)**
		**Male (%), *n* = 46**	**Female (%), *n* = 83**		
Alcohol	NO	33 (71.7)	60 (72.3)	93 (72.1)	0.019 (0.890)
	YES	13 (28.3)	23 (27.7)	36 (27.9)	
Smoke	NO	38 (82.6)	64 (77.1)	102 (79.1)	0.541 (0.610)
	YES	8 (17.4)	19 (22.9)	27 (20.9)	
TRADOMED + HAART	NO	39 (84.8)	64 (77.1)	103 (79.8)	0.659 (0.417)
	YES	7 (15.2)	19 (22.9)	26 (20.2)	

*n, distribution;*

Y *Yates correction*.

**Table 2:** The CD4 cells count and HAART duration for substance use categories.

**Groups**	**[Mean** **±** **S.D (median)]**		**Comparison of medians**	
	**Yes**	**No**	**W**	***P*-value**
**CD4 before HAART in cells/mm** ^**3**^				
Alcohol use	312.08 ± 106.12 [285.0]	287.48 ± 87.29 [265.0]	1,892.5	0.253
Smoking	308.52 ± 116.59 [252.0]	290.60 ± 86.21 [271.0]	1,413	0.837
Trado-medicine use	289.62 ± 99.99 [250.5]	295.54 ± 91.84 [275.0]	1,196	0.403
**HAART duration in months**				
Alcohol use	17.25 ± 4.62 [17.0]	17.75 ± 4.24 [16.5]	1,576.5	0.609
Smoking	18.44 ± 4.34 [17.0]	17.39 ± 4.55 [16.0]	1,599.5	0.197
Trado-medicine use	17.08 ± 4.25 [15.5]	17.75 ± 4.58 [17.0]	1,243.5	0.576

*S.D, Standard deviation; W, Mann-Whitney (Wilcoxon) value*.

The mean AST (YES = 39.23 ± 6.11 U/l, NO = 22.81 ± 3.42 U/l; *P* < 0.001), ALT (YES = 45.21 ± 6.22 U/l, NO = 29.62 ± 3.85; *P* < 0.001), and ALP (YES = 137.453 ± 2.20 U/l, NO = 132.01 ± 2.29 U/l; *P* = 0.007) values were significantly higher in patients that take alcohol. Similar results were obtained for patients who smoke, as the mean AST (YES = 47.17 ± 4.64 U/l, NO = 22.16 ± 3.21 U/l; *P* < 0.001). ALT (YES = 52.5119 ± 5.31 U/l, NO = 29.06 ± 3.60; *P* < 0.001) and ALP (YES = 138.0 ± 2.82 U/l, NO = 132.35 ± 2.11 U/l; *P* = 0.011) in the smokers group were significantly higher. For patients who take traditional remedies while on HAART, except for the AST values (YES = 33.88 ± 7.61 U/l, NO = 25.76 ± 3.55 U/l), which was significantly higher in patients on traditional remedies (*P* = 0.045), the ALT (YES = 39.53 ± 8.06 U/l, NO = 32.57 ± 3.84 U/l) and ALP (YES = 134.40 ± 4.00 U/l, NO = 133.31 ± 2.04 U/l) were not significantly different (*P* > 0.05) (Table 3).

**Table 3:** Liver enzyme levels, and test of mean difference in substance use (yes/no) groups.

**Variable**	**Statistical summary**		***T*** **-test of mean difference**	
**Alcohol use**	**NO (*n* = 93), Mean [95% CI]**	**YES (*n* = 36), Mean [95% CI]**	***T*-value**	***P*-value**
AST (U/l)	22.81 ± 3.42 [19.388, 26.236]	39.23 ± 6.11 [33.121, 45.3485]	−4.911	<0.001
ALT (U/l)	29.62 ± 3.85 [25.769, 33.471]	45.21 ± 6.22 [38.996, 51.432]	−4.269	<0.001
ALP (U/l)	132.01 ± 2.29 [129.723, 134.305]	137.45 ± 2.20 [135.254, 139.651]	−2.753	0.007
**Smoking**	**NO (*****n*** **=** **102), Mean [95% CI]**	**YES (*****n*** **=** **27), Mean [95% CI]**		
AST (U/l)	22.16 ± 3.21 [18.953, 25.366]	47.17 ± 4.64 [42.533, 51.815]	−7.458	<0.001
ALT (U/l)	29.06 ± 3.60 [25.468, 32.661]	52.51 ± 5.31 [47.198, 57.825]	−4.269	<0.001
ALP (U/l)	132.35 ± 2.11 [130.239, 134.453]	138.0 ± 2.82 [135.192, 140.83]	−2.593	0.011
**TRADOMED** **+** **HAART**	**NO (*****n*** **=** **103), Mean [95% CI]**	**YES (*****n*** **=** **26), Mean [95% CI]**		
AST (U/l)	25.76 ± 3.547 [22.210, 29.305]	33.88 ± 7.61 [26.270, 41.496]	−2.024	0.045
ALT (U/l)	32.57 ± 3.84 [28.7308, 36.4077]	39.53 ± 8.06 [31.473, 47.586]	−1.610	0.110
ALP (U/l)	133.31 ± 2.04 [131.268, 135.355]	134.40 ± 4.00 [130.406, 138.401]	−0.481	0.631

*AST, Aspartate Aminotransferase; ALT, Alanine Transaminase; ALP, Alkaline Phosphatase; C.I., Confidence Interval*.

**Figure 5:**
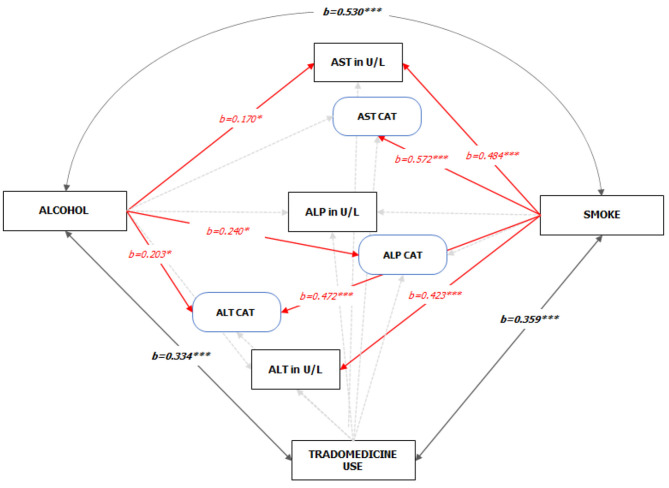
Standardized Pathways (z-statistic) to the enzyme levels of HIV-infected adult patients on HAART (See Figure A1.1 Supplementary Material for the unedited SPSS-Amos output). Note: 1. Red and faint ash colors indicate the significant and non-significant pathways; 2. *p < 0.05; ***p < 0.001; 3. GFI = 0.959, CFI = 0.985; 4. Sex, CD4 count on starting HAART, and Duration on HAART were statistically controlled.

For the path analysis, the GFI and the Bentler's CFI were 0.959 and 0.985, respectively, which reflects a satisfactory model fit (Chi-square_[df=42]_ = 65.903, *P* = 0.011). The reconstructed path diagram along with the standardized regression coefficients are depicted in Figure 5. The results from the model provided a partial support for our hypotheses. Specifically, alcohol was only associated with an elevated AST (*b* = 0.170, *p* = 0.05), while smoking was associated with a higher AST (*b* = 0484, *p* < 0.01) and ALT (*b* = 0.423, *p* < 0.01) values. In addition, alcohol use was associated with an abnormal ALT (*b* = 0.24, *p* < 0.05) and ALP (*b* = 0.203, *p* < 0.05), while smoking was associated with an abnormal AST (*b* = 0.572, *p* < 0.05). The standardized (z-statistic) and unstandardized direct effects of substance use on liver enzyme is presented in Table 4. The correlation and associations between the estimator and manifest variables can be seen in Figure A1.4.

**Table 4:** Standardized (z-statistic) and unstandardized direct effects of substance use on liver enzyme.

**Model variables**	**Standardized**			**Unstandardized**		
	**Alcohol**	**Smoke**	**Tradomed**	**Alcohol**	**Smoke**	**Tradomed**
ALP category	0.240	0.170	−0.036	0.242	0.189	−0.041
ALT category	0.193	0.472	−0.073	0.203	0.548	−0.086
AST category	0.151	0.572	−0.018	0.152	0.633	−0.02
ALP level	0.173	0.156	−0.072	4.006	3.982	−1.852
ALT level	0.154	0.423	−0.062	6.769	20.577	−3.057
AST level	0.170	0.484	−0.053	6.951	21.808	−2.407

## Discussion

The rise in HAART-induced hepatotoxicity raises a serious concern during the treatment and care of HIV/AIDS patients. On the other hand, patients who engage in anti-healthy social behaviors (36–39) do have poorer outcomes in HIV management (43–45), and substance abuse is prevalent among people with HIV (67, 68). Studies have documented, independently, that HAART (49–55) and certain social behaviors such as alcohol intake, smoking, and use of herbal remedies (40–42) have significant effects on the liver and may lead to mild to severe liver toxicity, and even death. The increase in the enzyme levels of HIV-treatment naïve patients (82) and those on HAART is well-documented (52–55). This study noted that the enzyme levels for a large proportion were just slightly above the normal limits; thus, suggesting that majority of the population were within mild to moderate enzyme elevations (54, 57).

In this study, more patients consumed alcohol than smoking or use of trado-medicine, with no sex-associated difference. The region is known for a high prevalence of alcohol consumption, smoking, and herbal remedy use (83, 84). Only 17.8% of the HIV-HAART patients presented with normal enzyme levels, suggesting that about 83.2% of the study population had one or more transaminase abnormality. In a 2007 study of HIV patients in UPTH, hepatic injuries (14.5%-cholestatic and 85.5%-hepatocellular) were common (82). Almost half (45.7%) of the study population had abnormal ALP levels independent of abnormal AST and ALT levels; thus, making it the most common enzyme abnormality, even among HIV-infected patients on HAART who do not use substances. Previous studies reported an elevated and abnormal ALP level among HIV patients on ART (25, 55, 85, 86).

Patients who take alcohol or smoke had a significantly higher mean AST, ALT, and ALP values compared to patients who do not take alcohol or smoke. Significant differences in the mean values for trado-medicine users were only indicative for AST. The direct association of substance use to liver enzymes levels was only partially indicative with alcoholism and smoking, but not trado-medicine, playing a low to moderate role in liver enzymes elevation and abnormality. The model showed that elevated AST levels and abnormal ALT were associated with alcohol use and smoking, while ALP abnormality was only related to alcohol use. Studies found that alcohol consumption was significantly associated with a raised ALT level in HIV-infected patients on ART at Hospitals in Ethiopia (87) and Switzerland (88). Although trado-medicine use was not associated with enzyme levels abnormalities; however, the study found an inverse relationship. The strong correlation between smoking and drinking and the fact that abnormal AST and ALT levels were higher among smokers is a pointer to possible additive effects. Studies have reported that social drinking is common in the southern part of Nigeria (83, 84) and a majority of social smokers take alcohol, with a well-documented supra-additive effect of alcohol on liver enzymes levels (70–73). Additionally, those who smoke or drink were more likely to develop one of the three enzyme abnormalities than those who use trado-medicine. From the analysis, alcohol drinkers and smokers were, in average, 20 and 6 times, respectively, more likely to have at least one liver enzyme abnormality than those who do not use such substances.

It is often difficult to precisely estimate the effect of substance use on medication because of the unpredictable nature of the associated risk or severity of substance-related toxicities and drug-substance interactions (69). The exact mechanism through which HIV medications are capable of causing liver enzyme elevations is still being evaluated and suggested to be complex because there is no clear explanation to the extent of damage the individual drugs induce or contribute to hepatotoxicity (89). However, because alcoholism and smoking are known to impact the liver through inflammatory pathways (72, 73), the hepatoxicities from an adverse drug reaction to HARRT (90–94) could be potentiated as a result of the metabolic pressures and supra-additive effects on the livers.

In the studied population, alcohol use was associated with abnormal ALT and ALP values, while smoking was associated with abnormal AST and ALP values. Alcohol was only associated with a raised AST level, while increased AST and ALT levels were factored on smoking. Trado-medicine was not a significant factor in enzymes level elevation or any abnormality. There was a stronger association between alcohol use and smoking than alcohol use and trado-medicine use and smoking and trado-medicine use.

## Conclusion

Among the studied HIV-infected adult patients on HAART, ALP abnormality was the most common, and there is a close association between elevated ALT and AST levels, without an elevated ALP level. Alcoholism and smoking, but not trado-medicine use, were directly associated to elevated ALT and AST levels and at least one or more abnormal transaminases.

The odds of developing one or more liver enzyme abnormality are against HIV patients who smoke, drink, or do both when on HAART therapy, and the possible explanation to this increased risk among alcohol users and smokers could be associated with the metabolic pressures and supra-additive effects on the livers.

### Limitation

Some limitations were observed and should be noted. The study recognized the small sample as a result of the population of interest (HIV-infected adults without comorbidity on HAART) and sincerely recommends a larger population for a definitive finding. The present study relied on self-reported measures for substance use using closed-ended questions (Yes/No), which may be problematic in terms of their reliability and validity; however, the DAST-10 scale for drug abuse uses such method and provides valid and reliable estimates (95). The study did not take into account the class of HAART used by the patients and, thus, could not evaluate the class-specific difference. Therefore, generalizing the findings may be difficult. The study recommends that future research should examine the pathways in more drug classes and sociocultural diversities in a larger population.

## Data Availability Statement

The datasets presented in this study can be found in online repositories. The names of the repository/repositories and accession number(s) can be found at: https://dataverse.harvard.edu/dataset.xhtml?persistentId=doi:10.7910/DVN/FJO2YX.

## Ethics Statement

The studies involving human participants were reviewed and approved by The Research Ethics Committee of University of Port Harcourt. Reference number: UPH/CEREMAD/REC/19. The patients/participants provided their written informed consent to participate in this study.

## Author Contributions

CA and EA conceptualized the research and supervised the data collection at the site. CA, EA, T-OJ, and IU drafted the initial research concept. AQ, SA, AA, JO, and GB drafted the sample collection, chemo-analytical protocols, and reviewed the final draft for ethical approval, EA, JO, T-OJ, IU, AQ, and SA managed, analyzed, and interpreted the data. IU, AA, JO, and GB provided an extensive literature review, which was scaled down by all authors. All authors proof-read the final manuscript.

## Conflict of Interest

The authors declare that the research was conducted in the absence of any commercial or financial relationships that could be construed as a potential conflict of interest.
