# Supplementary material for: Substance Use, Highly Active Antiretroviral Therapy, and Liver Enzymes: Evidence From a Cross-Sectional Study of HIV-Infected Adult Patients Without Comorbidities on HAART in the University of Port Harcourt Teaching Hospital
Source: Front Reprod Health. 2021 Jun 28;3:664080. doi: 10.3389/frph.2021.664080 (PMC9580740; doi:10.3389/frph.2021.664080)
Supplement: Supplementary file 1 [file Data_Sheet_1.docx]

# **APPENDICES**


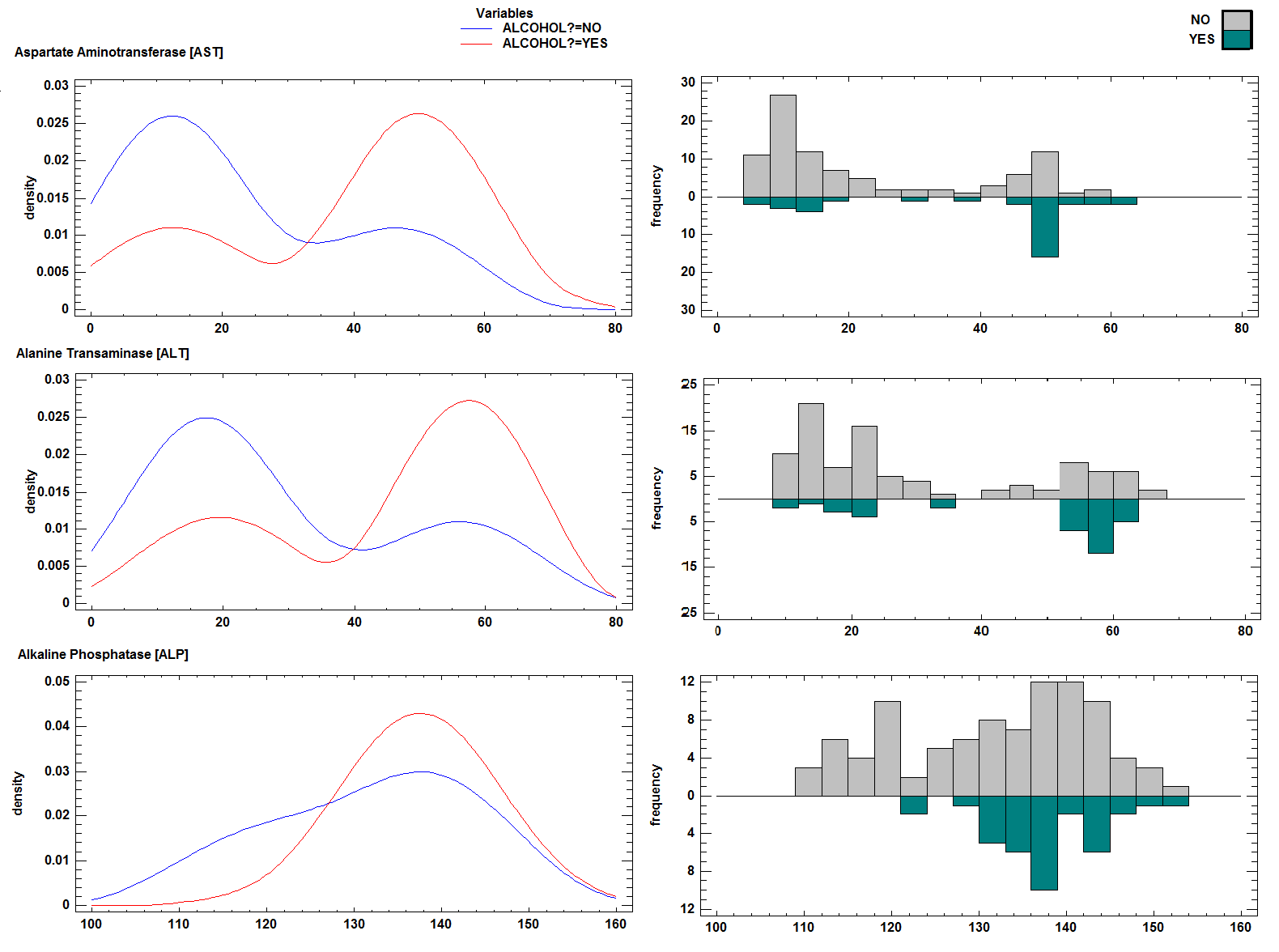


**Figure 1.1:** Alcohol consumption and distribution of the AST, ALT, and ALP among HIV-infected patients on HAART


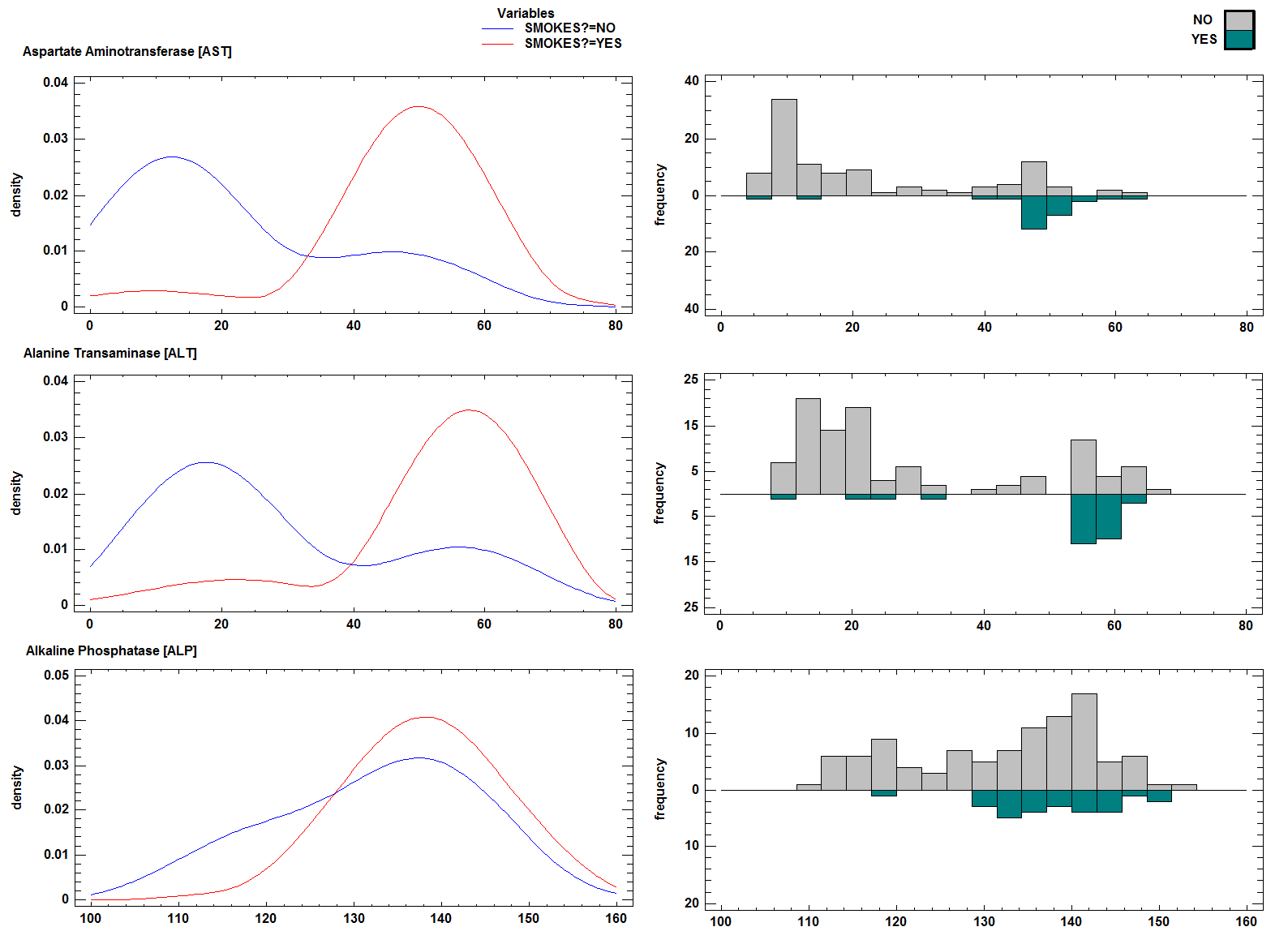


**Figure A1.2:** Smoking and distribution of the AST, ALT, and ALP among HIV-infected patients on HAART


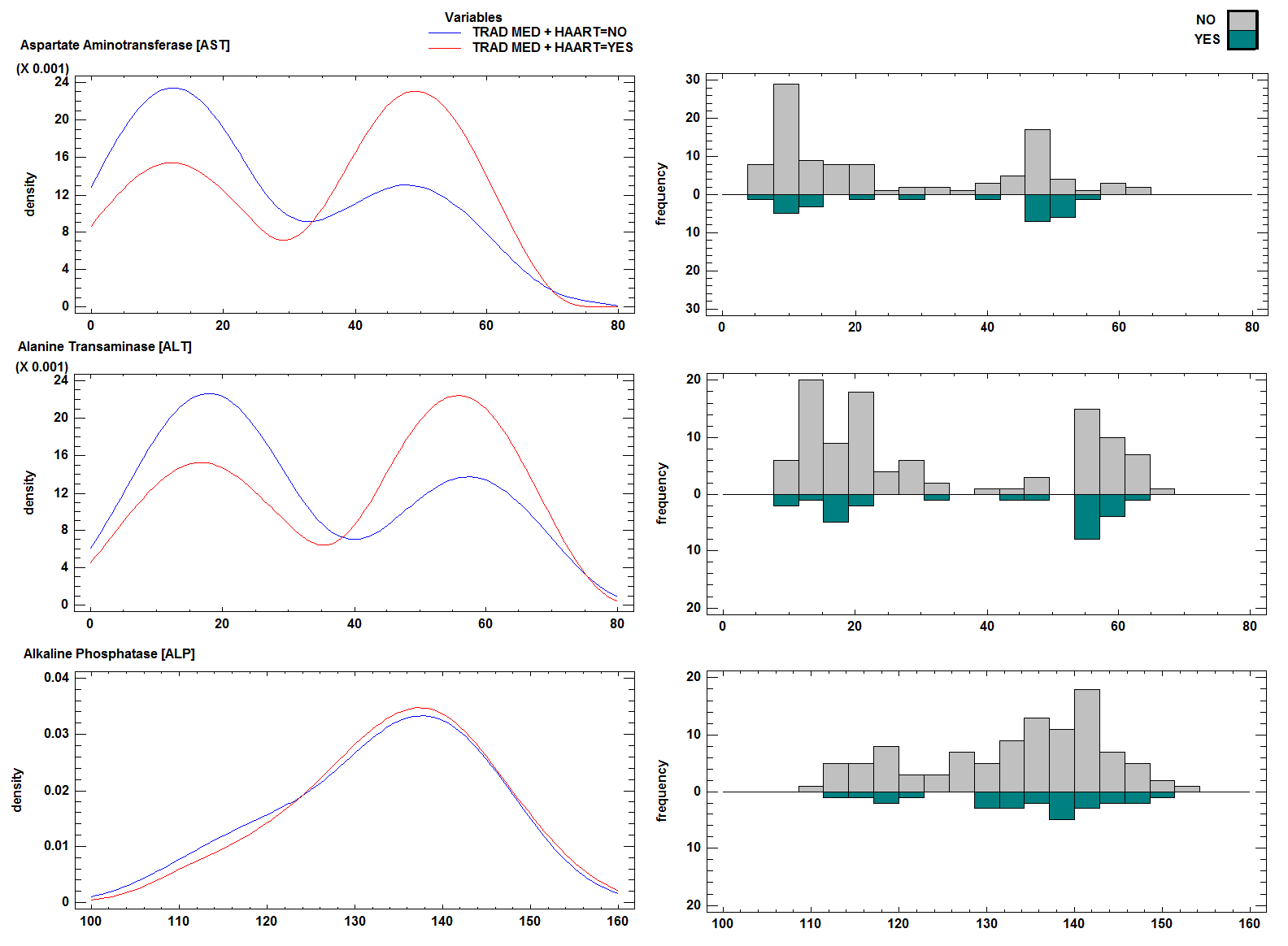


**Figure A1.3:** Tradomedicine and distribution of the AST, ALT, and ALP among HIV-infected patients on HAART


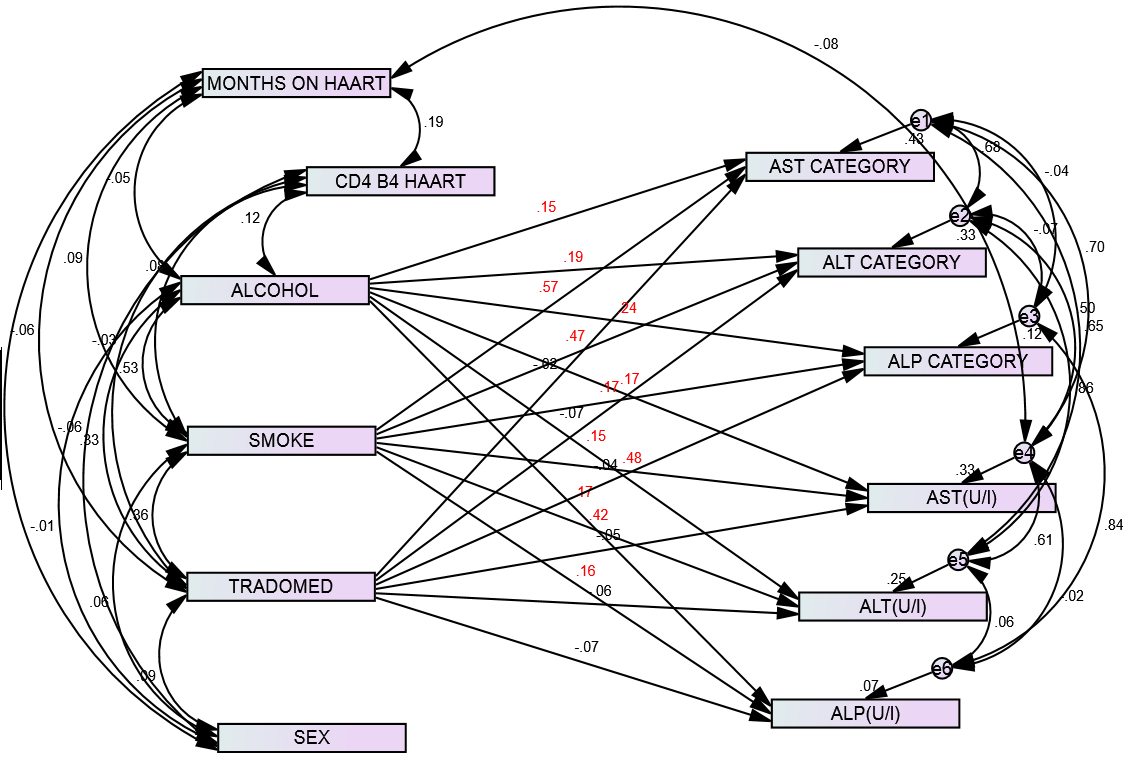


**Figure A1.4:** SPSS-Amos output for the SEM standardized pathways (z-statistic) to enzyme levels of HIV-infected adult patients on HAART

***Note:*** ***1.*** *Red colours indicates low to moderate estimates;* ***2.*** *GFI=0.959, CFI=0.985;* ***3.*** *Sex, CD4 count on starting HAART, and Duration on HAART were statistically controlled.*

**Table A1.1:** Regression weight and standardized (z-statistics) estimates in the SEM analysis

| **Variable Relationship** | | | **Estimate** | **Z-Estimate** | **S.E.** | **C.R.** | **P** |
| --- | --- | --- | --- | --- | --- | --- | --- |
| AST | <--- | ALCOHOL | 6.951 | 0.17 | 3.545 | 1.961 | 0.05 |
| AST | <--- | SMOKE | 21.808 | 0.484 | 3.948 | 5.524 | *** |
| AST | <--- | TRADOMED | -2.407 | -0.053 | 3.603 | -0.668 | 0.504 |
| ALT | <--- | ALCOHOL | 6.769 | 0.154 | 4.044 | 1.674 | 0.094 |
| ALP | <--- | ALCOHOL | 4.006 | 0.173 | 2.36 | 1.697 | 0.09 |
| ALP | <--- | SMOKE | 3.982 | 0.156 | 2.629 | 1.515 | 0.13 |
| ALT | <--- | TRADOMED | -3.057 | -0.062 | 4.11 | -0.744 | 0.457 |
| ALP | <--- | TRADOMED | -1.852 | -0.072 | 2.399 | -0.772 | 0.44 |
| ALT | <--- | SMOKE | 20.577 | 0.423 | 4.504 | 4.569 | *** |
| AST_Cat | <--- | ALCOHOL | 0.152 | 0.151 | 0.08 | 1.892 | 0.058 |
| AST_Cat | <--- | SMOKE | 0.633 | 0.572 | 0.089 | 7.089 | *** |
| ALT_Cat | <--- | SMOKE | 0.548 | 0.472 | 0.102 | 5.381 | *** |
| ALT_Cat | <--- | ALCOHOL | 0.203 | 0.193 | 0.091 | 2.225 | 0.026 |
| AST_Cat | <--- | TRADOMED | -0.02 | -0.018 | 0.081 | -0.247 | 0.805 |
| ALT_Cat | <--- | TRADOMED | -0.086 | -0.073 | 0.093 | -0.926 | 0.355 |
| ALP_Cat | <--- | TRADOMED | -0.041 | -0.036 | 0.102 | -0.4 | 0.689 |
| ALP_Cat | <--- | ALCOHOL | 0.242 | 0.24 | 0.1 | 2.417 | 0.016 |
| ALP_Cat | <--- | SMOKE | 0.189 | 0.17 | 0.112 | 1.693 | 0.09 |

***Note:*** ***1.*** *Z-standardised score;* ***2.*** *S.E-Standard error; C.R-critical ratio; P-probability; ***p<0.001;* ***3.*** *Sex, CD4 count on starting HAART, and Duration on HAART were statistically controlled.*

**Table A1.2:** Covariance and correlations of variables in the SEM analysis

| **Variable Relationship** | | | | **Estimate** | | **r** | | **S.E.** | | **C.R.** | | **P** |
| --- | --- | --- | --- | --- | --- | --- | --- | --- | --- | --- | --- | --- |
| SMOKE | <--> | TRADOMED | 0.059 | | 0.359 | | 0.015 | | 3.823 | | *** | |
| ALCOHOL | <--> | TRADOMED | 0.060 | | 0.334 | | 0.017 | | 3.581 | | *** | |
| ALCOHOL | <--> | SMOKE | 0.097 | | 0.530 | | 0.018 | | 5.295 | | *** | |
| ALCOHOL | <--> | SEX | -0.001 | | -0.006 | | 0.019 | | -0.066 | | 0.947 | |
| TRADOMED | <--> | SEX | 0.018 | | 0.092 | | 0.017 | | 1.032 | | 0.302 | |
| SMOKE | <--> | SEX | 0.013 | | 0.065 | | 0.017 | | 0.731 | | 0.465 | |
| ALCOHOL | <--> | MONTHS_HAART | -0.101 | | -0.050 | | 0.179 | | -0.565 | | 0.572 | |
| ALCOHOL | <--> | CD4_Prior | 4.949 | | 0.119 | | 3.705 | | 1.336 | | 0.182 | |
| SMOKE | <--> | MONTHS_HAART | 0.174 | | 0.095 | | 0.163 | | 1.070 | | 0.285 | |
| SMOKE | <--> | CD4_Prior | 2.966 | | 0.079 | | 3.347 | | 0.886 | | 0.376 | |
| TRADOMED | <--> | MONTHS_HAART | -0.108 | | -0.060 | | 0.160 | | -0.674 | | 0.500 | |
| TRADOMED | <--> | CD4_Prior | -0.954 | | -0.026 | | 3.292 | | -0.290 | | 0.772 | |
| SEX | <--> | CD4_Prior | -2.449 | | -0.055 | | 3.935 | | -0.622 | | 0.534 | |
| SEX | <--> | MONTHS_HAART | -0.263 | | -0.122 | | 0.191 | | -1.375 | | 0.169 | |
| MONTHS_HAART | <--> | CD4_Prior | 77.583 | | 0.185 | | 37.351 | | 2.07 | | 0.038 | |
| e2 | <--> | e1 | 0.089 | | 0.678 | | 0.014 | | 6.379 | | *** | |
| e2 | <--> | e3 | -0.011 | | -0.067 | | 0.006 | | -1.907 | | 0.056 | |
| e1 | <--> | e3 | -0.005 | | -0.037 | | 0.006 | | -0.933 | | 0.351 | |
| e5 | <--> | e4 | 156.131 | | 0.608 | | 26.497 | | 5.892 | | *** | |
| e5 | <--> | e6 | 10.824 | | 0.063 | | 5.959 | | 1.816 | | 0.069 | |
| e4 | <--> | e6 | -3.294 | | -0.022 | | 6.128 | | -0.537 | | 0.591 | |
| e3 | <--> | e6 | 3.567 | | 0.842 | | 0.484 | | 7.369 | | *** | |
| e2 | <--> | e5 | 5.724 | | 0.864 | | 0.767 | | 7.462 | | *** | |
| e1 | <--> | e5 | 3.803 | | 0.654 | | 0.611 | | 6.228 | | *** | |
| e2 | <--> | e4 | 2.910 | | 0.501 | | 0.571 | | 5.092 | | *** | |
| e1 | <--> | e4 | 3.576 | | 0.702 | | 0.549 | | 6.516 | | *** | |
| e4 | <--> | MONTHS_HAART | -5.256 | | -0.078 | | 3.787 | | -1.388 | | 0.165 | |

***Note:*** ***1.*** *Z-standardised score;* ***2.*** *r-correlation;* *S.E-standard error; C.R-critical ratio; P-probability; ***p<0.001;* ***3.*** *e-error*
